# A multi-omics digital research object for the genetics of sleep regulation

**DOI:** 10.1038/s41597-019-0171-x

**Published:** 2019-10-31

**Authors:** Maxime Jan, Nastassia Gobet, Shanaz Diessler, Paul Franken, Ioannis Xenarios

**Affiliations:** 1 Centre for Integrative Genomics, University of Lausanne, Lausanne, Switzerland; 2 Vital-IT, Swiss Institute of Bioinformatics, Lausanne, Switzerland; 3 Ludwig Cancer Research/CHUV-UNIL, Lausanne, Switzerland; 4 Health 2030 Genome Center, Geneva, Switzerland

**Keywords:** Systems analysis, Communication and replication, Mouse, Sleep disorders, Data integration

## Abstract

With the aim to uncover the molecular pathways underlying the regulation of sleep, we recently assembled an extensive and comprehensive systems genetics dataset interrogating a genetic reference population of mice at the levels of the genome, the brain and liver transcriptomes, the plasma metabolome, and the sleep-wake phenome. To facilitate a meaningful and efficient re-use of this public resource by others we designed, describe in detail, and made available a Digital Research Object (DRO), embedding data, documentation, and analytics. We present and discuss both the advantages and limitations of our multi-modal resource and analytic pipeline. The reproducibility of the results was tested by a bioinformatician not implicated in the original project and the robustness of results was assessed by re-annotating genetic and transcriptome data from the mm9 to the mm10 mouse genome assembly.

## Abstract (summary)

**Table:** 

Design Type(s)	transcription profiling design • molecular interaction analysis objective • metabolic network analysis objective • data integration objective
Measurement Type(s)	behavioral phenotype • transcription profiling assay • metabolomic assay • physical activity
Technology Type(s)	electrical potential recording method • RNA sequencing • liquid chromatography-tandem mass spectrometry • Infrared Spectrometry
Factor Type(s)	experimental condition
Sample Characteristic(s)	Mus musculus • Cortex • Liver • Blood

Machine-accessible metadata file describing the reported data (ISA-Tab format)

## Background & Summary

A good night’s sleep is essential for optimal performance, wellbeing and health. Chronically disturbed or curtailed sleep can have long-lasting adverse effects on health with associated increased risk for obesity and type-2 diabetes^1^.

To gain insight into the molecular signaling pathways regulating undisturbed sleep and the response to sleep restriction in the mouse, we performed a population-based multi-level screening known as *systems genetics*^2^. This approach allows to chart the molecular pathways connecting genetic variants to complex traits through the integration of multiple *omics datasets such as transcriptomics, proteomics, metabolomics or microbiomes^3^.

We built a systems genetics resource based on the BXD panel, a population of recombinant inbred lines of mice^4^, that has been used for a number of complex traits and *omics screening such as brain slow-waves during NREM sleep^5^, glucose regulation^6^, cognitive aging^7^ and mitochondria proteomics^8^.

**Fig. 1:**
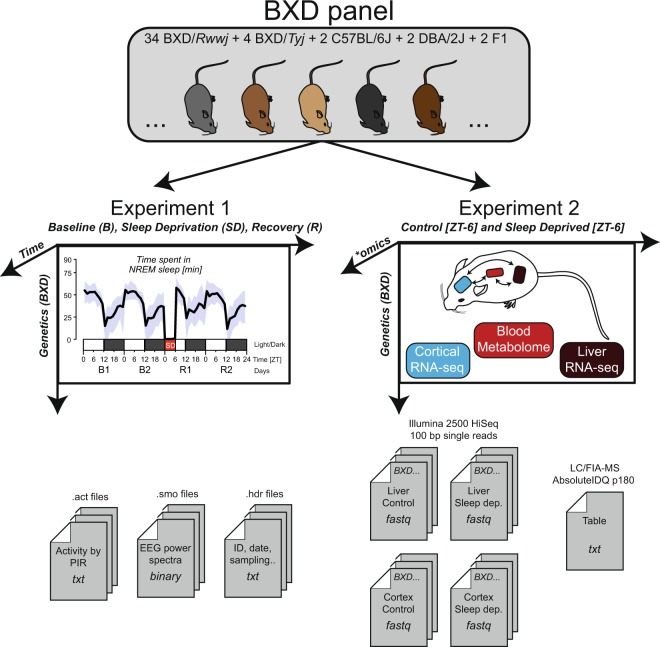
Data generation. The behavioral/EEG end-phenotypes of the BXD mouse panel were quantified in Experiment 1. Mice were recorded for 4 days: 2 days of baseline (B1 & B2), followed by 6 h of sleep deprivation (SD) and 2 days of recovery (R1 & R2). EEG spectral composition was written in .*smo* files, activity in .*act* files and meta-data in .*hdr* files. Blood metabolomics, liver transcriptomics and cortical transcriptomics were quantified in Experiment 2. ‘Control’ and ‘Sleep deprived’ batches were sampled at a single time point: ZT6 (i.e. directly after sleep deprivation for the ‘sleep deprived’ batch). Transcriptomics was performed on pooled sampled per BXD strains. For blood metabolomics, metabolite quantification was performed for each BXD replicates. Adapted from^2^.

We phenotyped 34 BXD/RwwJ inbred lines, 4 BXD/TyJ, 2 parental strains C57BL6/J and DBA/2 J and their reciprocal F1 offspring. Mice of these 42 lines were challenged with 6 h of sleep deprivation (SD) to evaluate the effects of insufficient sleep on sleep-wake behavior and brain activity (electroencephalogram or EEG; Fig. 1, Experiment 1) and, on gene expression and metabolites (Fig. 1, Experiment 2). For Experiment 1 we recorded the EEG together with muscle tone (electromyogram or EMG) and locomotor activity (LMA) continuously for 4 days. Based on the EEG/EMG signals we determined sleep-wake state [wakefulness, rapid-eye movement (REM) sleep, and non-REM (NREM) sleep] as well as the spectral composition of the EEG signal as end phenotypes. For Experiment 2 we quantified mRNA levels in cerebral cortex and liver using illumina RNA-sequencing and performed a targeted metabolomics screen on blood using Biocrates p180 liquid chromatography (LC-) and Flow injection analysis (FIA-) coupled with mass spectrometry (MS). These transcriptome and metabolome data are regarded as intermediate phenotypes linking genome information to the sleep-wake related end phenotypes.

The keystone of systems genetics is data integration. Accordingly, the scientific community can benefit from data sharing strategies that facilitate the integration of datasets among research groups. However, reliable methods for data integration are needed and require a broad range of expertise such as in mathematical and statistical models^9^, computational methods^10^, visualization strategies^11^, and deep understanding of complex phenotypes. Therefore, data sharing should not be limited to the dataset *per se* but also to analytics in the form of analysis workflows, code, interpretation of results, and meta-data^12^. The concept of a Digital Research Object (DRO) was proposed to group dataset and analytics into one united package^13^. Various guidelines have been suggested to address the challenges of sharing such DRO with the goal to improve and promote the human and computer knowledge sharing, like the FAIR (Findable, Accessible, Interoperable, Reusable) principles proposed by FORCE 11^14^ or by the DB2K (Big Data to Knowledge) framework. These guidelines concern biomedical workflow, meta-data structures and computer infrastructures facilitating the reusability and interoperability of digital resources^15^. Although such guidelines are often described and applied in the context of single data-type assays, they can be challenging to achieve for trans-disciplinary research projects such as systems genetics, in which multiple data types, computer programs, references and novel methodologies need to be combined^16^. Moreover, applying these principles can also be discouraging because of the time required for new working routines to become fully reproducible^17^ and because only few biomedical journals have standardized and explicit data-sharing^18^ or reproducibility^19^ policies. Nonetheless, DROs are essential for scientific reliability^20^, and can save time if a dataset or methods specific to a study need to be reused or improved by different users such as colleagues at other institutes, new comers to the lab, or at long-term yourself.

We here complement our previous publication^2^ by improving the raw and processed data availability. We describe in more details the different bioinformatics steps that were applied to analyze this resource and improve the analytical pipeline reproducibility by generating *R* reports and provide code. Finally, we assess the reproducibility of our bioinformatic pipeline from the perspective of a new student in bioinformatics that recently joined the group, and the robustness of the results by changing both the mouse reference genome and the RNA-seq reads alignment to new standards.

## Methods

The methods detailed below are an expanded version of the methods described in our related paper^2^. Appreciable portions are reproduced verbatim to deliver a complete description of the data and analytics with the aim to enhance reproducibility.

Experiment 1 and Experiment 2 (Fig. 1) were approved by the veterinary authorities of the state of Vaud, Switzerland (SCAV authorization #2534).

### Animals, breeding, and housing conditions

34 BXD lines originating from the University of Tennessee Health Science Center (Memphis, TN, United States of America) were selected for Experiment 1 and Experiment 2. These lines were randomly chosen from the newly generated advanced recombinant inbred line (ARIL) RwwJ panel^4^, although lines with documented poor breeding performance were not considered. 4 additional BXD RI strains were chosen from the older *TyJ* panel for reproducibility purposes and were obtained directly from the Jackson Laboratory (JAX, Bar Harbor, Maine). The names used for some of the BXD lines have been modified over time to reflect genetic proximity. Online-only Table 1 lists the BXD line names we used in our files alongside the corresponding current JAX names and IDs. In our analyses, we discarded the BXD63/RwwJ line for quality reasons (see Technical Validation) as well as the 4 older BXD strains that were derived from a different DBA/2 sub-strain, i.e. DBA/2Rj instead of DBA/2 J for RwwJ lines^21^. The methods below describe the remaining 33 BXD lines, F1 and parental strains.

Two breeding trios per BXD strain were purchased from a local facility (EPFL-SV, Lausanne, Switzerland) and bred in-house until sufficient offspring was obtained. The parental strains DBA/2 J (D2), C57BL6/J (B6) and their reciprocal F1 offspring (B6D2F1 [BD-F1] and D2B6F1 [DB-F1]) were bred and phenotyped alongside. Suitable (age and sex) offspring was transferred to our sleep-recording facility, where they were singly housed, with food and water available *ad libitum*, at a constant temperature of 25 °C and under a 12 h light/12 h dark cycle (LD12:12, fluorescent lights, intensity 6.6 cds/m^2^, with Zeitgeber time 0 (ZT0) and ZT12 designating light and dark onset, respectively). Male mice aged 11–14 week at the time of experiment were used for phenotyping, with a mean of 12 animals per BXD line among all experiments. Note that 3 BXD lines had a lower replicate number (n), with respectively BXD79 (n = 6), BXD85 (n = 5), and BXD101 (n = 4) because of poor breeding success. For the remaining 30 BXD lines, replicates were distributed as follows: for EEG/behavioral phenotyping (Experiment 1 in Fig. 1; mean = 6.2/line; 5 ≤ n ≤ 7) and for molecular phenotyping (Experiment 2 in Fig. 1; mean = 6.8/line; 6 ≤ n ≤ 9). Additionally, to control for the reproducibility of the outcome variables over the course of the experiment, parental lines were phenotyped twice—i.e., at the start (labeled in files as B61 and DB1) and end (labeled B62 and DB2) of the breeding and data-collecting phase, which spanned 2 years (March 2012–December 2013). To summarize, distributed over 32 experimental cohorts, 227 individual mice were used for behavioral/EEG phenotyping (Experiment 1) and 263 mice for tissue collection for transcriptome and metabolome analyses (Experiment 2), the latter being divided into sleep deprived (SD) and controls (“Ctr”; see Study design section below). We put in an effort to distribute the lines across the experimental cohorts so that biological replicates of 1 line were collected/recorded on more than 1 occasion while also ensuring that an even number of mice per line was included for tissue collection so as to pair SD and “Ctr” individuals within each cohort (for behavioral/EEG phenotyping, each mouse serves as its own control).

### Study design and sleep deprivation

The study consisted of 2 experiments, i.e., Experiments 1 and 2 (Fig. 1). Animals of both experiments were maintained under the same housing conditions. Animals in Experiment 1 underwent surgery and, after a > 10 days recovery period, electroencephalography (EEG), electromyography (EMG) and locomotor activity (LMA) were recorded continuously for a 4-day period starting at ZT0. The first 2 days were considered Baseline (B1 and B2). The first 6 hours of Day 3 (ZT0–6), animals were sleep deprived (SD) in their home cage by “gentle handling” referring to preventing sleep by changing litter, introducing paper tissue, presenting a pipet near the animal, or gently tapping the cage. Experimenters performing the SD rotated every 1 or 2 hours (for more information, see^22^). The remaining 18 h of Day 3 and the entire Day 4 were considered Recovery (R1 and R2).

Half of the animals included in Experiment 2 underwent SD alongside the animals of Experiment 1. The other half was left undisturbed in another room (i.e., control or Ctr, also referred as Non Sleep Deprived or NSD). Both SD and “Ctr” mice of Experiment 2 were sacrificed at ZT6 (i.e., immediately after the end of the SD) for sampling of liver and cerebral cortex tissue as well as trunk blood. All mice were left undisturbed for at least 2 days prior to SD.

### Experiment 1: EEG/EMG and LMA recording and signal pre-processing

EEG/EMG surgery was performed under deep anesthesia. IP injection of Xylazine/Ketamine mixture (91/14.5 mg/kg, respectively) ensures a deep plane of anesthesia for the duration of the surgery (i.e., around 30 min). Analgesia was provided the evening prior and the 3 days after surgery with Dafalgan in the drinking water (200–300 mg/kg). Six holes were drilled into the cranium, 4 for screws to fix the connector with Adhesive Resin Cement, 2 for EEG electrodes. The caudal electrode was placed over the hippocampal structure and the rostral electrode was placed over the frontal cerebral cortex. Two gold-wire electrodes were inserted into the neck muscle for EMG recording (for details, see^22^). Mice were allowed to recover for at least 10 days prior to baseline recordings. EEG and EMG signals were amplified, filtered, digitized, and stored using EMBLA (Medcare Flaga, Thornton, CO, USA) hardware (A10 recorder) and software (Somnologica). Digitalization of the signal was performed as followed: the analog-to-digital conversion of the signal was performed at a rate of 2000 Hz, the signal was down sampled at 200 Hz, high-pass filter at 0.0625 Hz was applied to reject DC offset of the signal and a 50-Hz notch filter applied to reduce line artefacts. Signals were transformed by Discrete Fourier Transform (DFT) to yield power spectra between 0 and 100 Hz with a 0.25 frequency resolution using a 4-seconds time resolution (referred to as a 4 s “epoch”). EEG frequency bins with artefacts of known (line artefacts between 45–55 Hz) and unknown (75–77 Hz) source were removed from the average EEG spectra of all mice. Other specific 0.25 Hz bins containing artefacts (notably the 8.0, 16.0 and 32.0 Hz bins) of unknown source, were removed from individual mice based on the visual inspection of individual EEG spectra in each of the three sleep-wake states (i.e. wakefulness, REM sleep and NREM sleep). Power density in frequency bins deemed artefacted were estimated by linear interpolation. For details, see Pascal scripts in https://gitlab.unil.ch/mjan/Systems_Genetics_of_Sleep_Regulation.

LMA was recorded by passive infrared (PIR) sensors (Visonic, Tel Aviv, Israel) at 1-min resolution for the duration of the 4-day experiment, using ClockLab (ActiMetrics, IL, USA). Activity data were made available as .*act* files at Figshare^23^.

Offline, the sleep-wake states wakefulness, REM sleep, and NREM sleep were annotated on consecutive 4-second epochs, based on the EEG and EMG pattern (see Sleep-wake state annotation section). EEG/EMG power spectra and sleep-wake state annotation were made available as binary (.*smo*) files at Figshare^23^.

### Experiment 2: Tissue collection and preparation

Mice were sacrificed by decapitation after being anesthetized with isoflurane, and blood, cerebral cortex, and liver were collected immediately. The whole procedure took no more than 5 min per mouse. Blood was collected at the decapitation site into tubes containing 10 ml heparin (2 U/μl) and centrifuged at 4000 rpm during 5 min at 4 °C. Plasma was collected by pipetting, flash-frozen in liquid nitrogen, and stored at −80 °C until further use. Cortex and liver were flash-frozen in liquid nitrogen immediately after dissection and were stored at −140 °C until further use.

#### RNA extraction and pooling

For RNA extraction, frozen samples were homogenized for 45 seconds in 1 ml of QIAzol Lysis Reagent (Qiagen; Hilden, Germany) in a gentleMACS M tube using the gentleMACS Dissociator (Miltenyi Biotec; Bergisch Gladbach, Germany). Homogenates were stored at −80 °C until RNA extraction. Total RNA was isolated and purified from cortex using the automated nucleic acid extraction system QIAcube (Qiagen; Hilden, Germany) with the RNeasy Plus Universal Tissue mini kit (Qiagen; Hilden, Germany) and were treated with DNAse. Total RNA from liver was isolated and purified manually using the Qiagen RNeasy Plus mini kit (Qiagen; Hilden, Germany), which includes a step for effective elimination of genomic DNA. RNA quantity, quality, and integrity were assessed utilizing the NanoDrop ND-1000 spectrophotometer (Thermo scientific; Waltham, Massachusetts, USA) and the Fragment Analyzer (Advanced Analytical). The 263 mice initially sacrificed for tissue collection yielded 222 cortex and 222 liver samples of good quality.

Equal amounts of RNA from biological replicates (3 samples per strain, tissue, and experimental condition, except for BXD79, BXD85, and BXD101; see above under Animals, breeding, and housing conditions) were pooled, yielding 156 samples for library preparation. RNA-seq libraries were prepared from 500 ng of pooled RNA using the Illumina TruSeq Stranded mRNA reagents (Illumina; San Diego, California, USA) on a Caliper Sciclone liquid handling robot (PerkinElmer; Waltham, Massachusetts, USA).

#### RNA sequencing

**Fig. 2:**
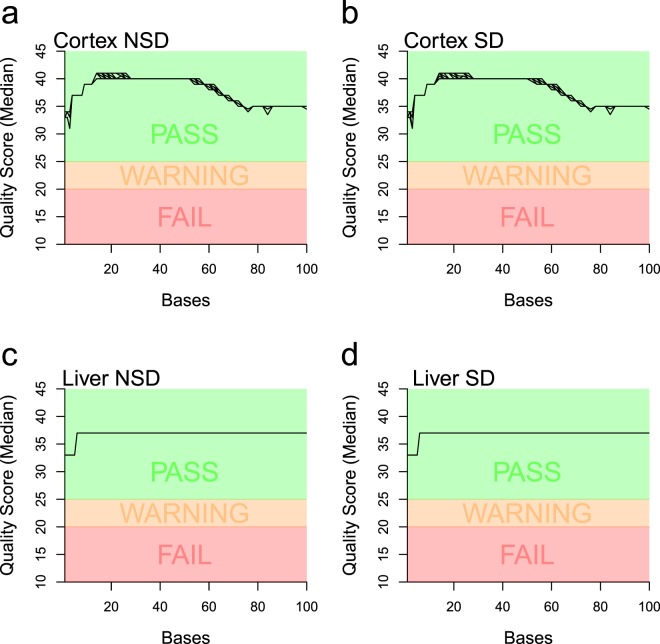
Median PHRED read quality per base for BXD RNA-sequencing. PHRED quality score based on illumina 1.9. (**a**) Samples from Cortex during control (NSD). (**b**) Samples from Cortex after sleep deprivation (SD). (**c**) Samples from Liver during control (NSD). (**d**) Samples from Liver after sleep deprivation (SD). Median score was computed using MultiQC^69^.

Libraries were sequenced on the Illumina HiSeq. 2500 using HiSeq SBS Kit v3 reagents, with cluster generation using the Illumina HiSeq PE Cluster Kit v3 reagents. A mean of 41 M 100 bp single-end reads were obtained (29 M ≤ n ≤ 63 M). Quality of sequences were evaluated using FastQC software (version 0.10.1) and reports made available here https://bxd.vital-it.ch/#/dataset/1. Figure 2 (a, b, c and d) shows the median Phred quality score per base among all samples reads for ‘Cortex Control’, ‘Cortex SD’, ‘Liver Control’ and ‘Liver SD’ respectively. Fastq files were made available at NCBI Gene Expression Omnibus^24^.

#### Targeted LC-MS metabolomics

Targeted metabolomics analysis was performed using flow injection analysis (FIA) and liquid chromatography/mass spectrometry (LC/MS) as described in^25,26^. To identify metabolites and measure their concentrations, plasma samples were analyzed using the AbsoluteIDQ p180 targeted metabolomics kit (Biocrates Life Sciences AG, Innsbruck, Austria) and a Waters Xevo TQ-S mass spectrometer coupled to an Acquity UPLC liquid chromatography system (Waters Corporation, Milford, MA, USA). The kit provided absolute concentrations for 188 endogenous compounds from 6 different classes, namely acyl carnitines, amino acids, biogenic amines, hexoses, glycerophospholipids, and sphingolipids. Plasma samples were prepared according to the manufacturer’s instructions. Sample order was randomized, and 3 levels of quality controls (QCs) were run on each 96-well plate. Data were normalized between batches, using the results of quality control level 2 (QC2) repeats across the plate (n = 4) and between plates (n = 4) using Biocrates METIDQ software (QC2 correction). Metabolites below the lower limit of quantification or the limit of detection, as well as above the upper limit of quantification, or with standards out of limits, were discarded from the analysis^26^. Out of the 188 metabolites assayed, 124 passed these criteria across samples and were used in subsequent analyses. No hexoses were present among the 124 metabolites. Out of the 256 mice sacrificed for tissue collection, 249 plasma samples were used for this analysis. An average of 3.5 animals (3 ≤ n ≤ 6) per line and experimental condition were used (except for BXD79, BXD85, and BXD101 with respectively 2, 1, and 1 animal/condition used; see above under Animals, breeding, and housing conditions). Note that in contrast to the RNA-seq experiment, samples were not pooled but analyzed individually. Mean metabolite levels per BXD line were made available at https://bxd.vital-it.ch/#/dataset/1 for details see intermediate files^27^.

#### Corticosterone quantification

In the same plasma samples, we determined corticosterone levels using an enzyme immunoassay (corticosterone EIA kit; Enzo Life Sciences, Lausanne, Switzerland) according to the manufacturer’s instructions. All samples were diluted 40 times in the provided buffer, kept on ice during the manipulation, and tested in duplicate. BXD lines were spread over multiple 96-well plates in an attempt to control for possible batch effects. In addition, a “control” sample was prepared by pooling plasma from 5 C57BL6/J mice. Aliquots of this control were measured along with each plate to assess plate-to-plate variability. The concentration was calculated in pg/ml based on the average net optical density (at λ = 405 nm) for each standard and sample.

Corticosterone level were made available on Figshare^27^.

### Bioinformatics pipeline

**Fig. 3:**
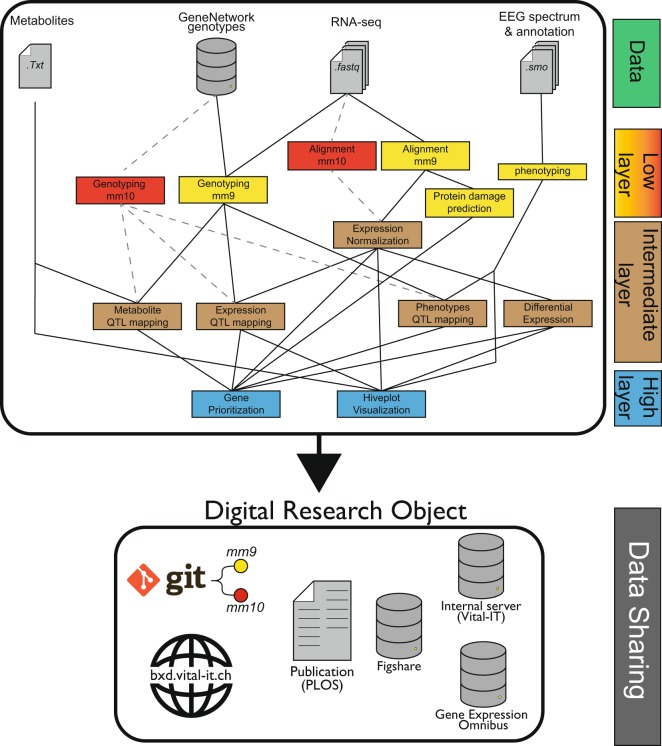
Summary of the bioinformatic analytical pipeline. Representation of the main bioinformatics methods used. Original analyses were performed using the mm9 mouse assembly (yellow). Results were also reproduced using the mm10 mouse assembly (red) and all downstream analyses. Layers represent the scripts organization on gitlab and available intermediate files.

To facilitate the interpretation of the complete bioinformatic workflow that was performed on this dataset, we here describe first our general strategy to construct an analytics pipeline with which we hope to improve reproducibility (Fig. 3). This strategy has some similarities with the recently published tool Qresp^28^ that facilitates the visualization of paper workflow. We then describe the specific methods used to analyze this dataset.

The analytics and input datasets were separated into 3 layers according to an increasing level of data abstraction (Fig. 3). This hierarchical structure of the workflow was particularly useful to identify steps downstream novel versions of a script or data (e.g. Figure 3, red) and simplify workflow description. The first *low-level* layer contains the procedures needed to reduce and transform the raw-data (i.e. RNA-seq reads, EEG/EMG signals) into an exploitable signal such as sleep phenotypes, gene expression, or mice genotypes by further analytical steps. This layer is characterized by long and computationally intensive procedures which required the expertise of different persons, each with their own working environment and preferred informatics language.

The *intermediate-level* layer contains some established analyses that could be performed on the data such as gene expression normalization followed by differential expression or Quantitative Trait Locus (QTL) mapping. With the scripts of this layer we explored the effects of sleep deprivation, genetic variations, as well as their interaction on EEG/behavioral phenotypes and intermediate phenotypes.

The *high-level* layer contains the novel integrative methods that we developed to prioritize genes driving sleep regulation and to visually represent the meta-dimensional multi-omics networks underlying sleep phenotypes.

### Standard and non-standard semantics

To improve the reproducibility and reusability of our workflow, we tried to prioritize standard semantics and established pipelines when applicable, such as the RNA-seq processing by STAR and htseq-count^29^. The use of curated symbols for genes nomenclature by RefSeq allowed a better semantic interoperability with other resources such as Uniprot protein ID using solutions like biomaRt^30^. We provided some of the references files used in these scripts, like the RefSeq.*gtf* reference file (see the Exome/RefSeq_20140129.gtf file in the DataSystemsGeneticsOfSleep_mm9.tar.gz file^27^, this file comes from UCSC table browser and was generated using RefSeq Reflat database on the 2014/01/29).

These annotations can be updated and possibly change the gene quantification with updated version or different genome reference.

However, the EEG/behavioral phenotyping procedure could not be performed by any standard computational workflow or common semantics as none exist. The nomenclature that was chosen in this case to generate unique phenotypic ID was a combination of the phenotype observed (e.g. EEG power during NREM sleep) and the features observed in this phenotype (e.g. delta band 1–4 Hz). These phenotypes were also present as file name and column name in our dataset^27^. Nevertheless, we mapped our phenotypes to the Human Phenotype Ontology (HPO)^31^ to help non-specialists to explore these traits and facilitate human-mouse data integration. These associations are not exact matches as most of the terms available in the HPO are disease oriented while our phenotypes should be considered as normal traits for inbred lines. The mapping can be found in the *General_Information*.*xlsx* file (https://bxd.vital-it.ch/#/dataset/1).

### Favor *R* and *Rmarkdown* reports for reproducible results

After data processing within the *low-level* layer, the effect of sleep deprivation, genotype, and their interaction were measured using various statistical models and computational methods. We chose to prioritize the programming language *R* as it was the best suited tool for the statistical analyses and for the generation of figures. Beside the advantages of a license-free and portable language, *R* was already recommended as main tool for systems genetics analysis^32^. Many available packages were particularly adapted for the systems genetics design, involving phenotype-genotype association (*r/qtl*), network analysis (*WGCNA*, *SANTA*, *igraph*), differential expression (*EdgeR*, *DESeq*, *limma*), bayesian network learning (*bnlearn*), visualization (*ggplot2*, *grid*), enrichment (*topGO*, *topAnat*) and parallel computing (*parallel*). Only a few analyses were performed using other softwares, principally for efficiency reasons in cis-/trans-eQTL analysis where the number of models to test was quite large^33,34^. R is one of the flagships of open science and reproducibility^35^ with a reviewable source code and the possibility of generating reports known as ‘*Rmarkdown*’ with 2 packages: *knitr*^36^ and *rmarkdown*^37^. This report format contains combination of code, figures, and comments within a single *markdown* document that can be easily converted into *pdf* or *html* format. Rmarkdown scripts were made available (https://gitlab.unil.ch/mjan/Systems_Genetics_of_Sleep_Regulation) and the reports in the form of.html document were made available together with the data^27^. To avoid the need to copy/paste some functions shared between *Rmarkdowns* but still display them in our reports, we used the *readLines()* function within Rmarkdown chunks. Finally, the use of the *sessionInfo()* function at the end of the document allowed to keep track of the packages versions and the environment variable used. Some of these Rmarkdown reports were generated on a remote cluster instead of the more traditional Rstudio environment, for more information on how to generate these Rmarkdown, see the Usage Notes.

### Workflow documentation

This systems genetics approach was an integrative project that implicated multiple collaborators, that each contributed to the final results, with their own working habit related to their area of expertise. For better reproducibility of the generated files, a critical goal was to keep track of the different files created, associated documents or analytical steps that were produced. For example, EEG/behavioral phenotypes could be found within many files and reports, from *low-level* to *high-level* layers, but their nomenclatures were still hard to interpret as mentioned above, for those not directly related to this project. A newcomer in this project should be able to easily recover the metadata document containing all the physiological phenotypes information (i.e. understand that a metadata document was created and where to find it or who to ask for it) and understand which scripts were used to produce these phenotypes. To establish what was exactly performed, we generated a documentation file containing the essential information and relationships between all the files, scripts, Rmarkdown, small workflow or database used in this project. This document describes the inputs/outputs needed and where to locate the information distributed among different persons or different directories on a digital infrastructure as presented in Fig. 3 but with more details to improve the reproducibility of the DRO^38^.

The markdown format was kept as it was easy to write/read by a human or to generate via a python script. This file was formatted into a simplified RDF-like triples structure, were each file-object (subject) was linked to information (object) by a property. This format allowed to use the following properties to describe each file-objects we had: The file-object name or identification, a brief description (i.e. about the software used or the data content), the file-object version, the input(s)/output(s), the associated documents, hyperlink(s) to remote database or citation, the location of the file-object on the project directory or archiving system, and the author(s) to contact for questions. These associations could be viewed as a graph to display the important files and pipelines used. This document was useful to understand how exactly the different files were generated, and to recover the scripts and input/output used, even after prolonged periods and to use them again, which permits for example, to reproduce data with novel or updated annotation files. Furthermore, if an error was detected within a script, the results and figures downstream that needed to be recomputed could be easily found. This documentation file (Documentation.html) was made available on gitlab (https://gitlab.unil.ch/mjan/Systems_Genetics_of_Sleep_Regulation).

### Data mining website

The DRO built for this systems genetics resource is constituted of the following collection: raw-data, processed data, Rmarkdown reports, results & interpretation, workflow, scripts, and metadata. To improve the reproducibility of our integrative visualization method (see HivePlots below), we provided some data-mining tools, a server to store some intermediate results, and a web application^39,40^. The home page of the web application displays the information for the NREM sleep gain during the 24 hours (in four 6-hour intervals) after sleep deprivation. Three data-mining tutorials were described on the website the web interface to: (i) mine a single phenotype, (ii) search for a gene, and (iii) compare hiveplots. Currently, no centralized repository exists containing all types of phenotypic data that were extracted within this project. This web-interface can, however be viewed as a hub for this DRO that became findable and accessible with a web-browser. With this web resource, we provided an advanced interactive interface for EEG/behavioral end-phenotypes and their associated intermediate phenotypes (variants, metabolites, gene expression). Compared to other web-resources for systems genetics like GeneNetwork where the principal focus is QTL mining, this interface provides an integrative view of this one dataset, with also data files and link to code to reproduce some of our analyses in the form of Rmarkdown, like the prioritization strategy.

### Low-level layer analyses

#### Sleep-wake state annotation

To assist the annotation of this extensive dataset (around 20 million 4 s epochs), we developed a semiautomated scoring system. The 4-day recordings of 43 mice (19% of all recordings), representing animals from 12 strains, were fully annotated visually by an expert according to established criteria^22^. Due to large between-line variability in EEG signals, even after normalization, a partial overlap of the different sleep-wake states remained, as evidenced by the absolute position of the center of each state cluster, which differed even among individuals of the same line (precluding the use of 1 “reference” mouse), even per line, to reliably annotate sleep-wake states for the others. To overcome this problem, 1 day out of 4 (i.e., Day 3 or R1, which includes the SD) was visually annotated for each mouse. These 4 seconds sleep-wake scores were used to train the semiautomatic scoring algorithm, which took as input 82 numerical variables derived from the analyses of EEG and EMG signals using frequency- (discrete Fourier transform [DFT]) and time-domain analyses performed at 1 second resolution. We then used these data to train a series of support vector machines (SVMs)^41^ specifically tailored for each mouse, using combinations of the 5 or 6 most informative variables out of the 82 input variables. The best-performing SVMs for a given mouse were then selected based on the upper-quartile performance for global classification accuracy and sensitivity for REM sleep (the sleep-wake state with the lowest prevalence) and used to predict sleep-wake states in the remaining 3 days of the recording. The predictions for 4 consecutive 1-s epochs were converted into 1 four-second epoch. Next, the results of the distinct SVMs were collapsed into a consensus prediction, using a majority vote. In case of ties, epochs were annotated according to the consensus prediction of their neighboring epochs. To prevent overfitting and assess the expected performance of the predictor, only 50% of the R1 manually annotated data from each mouse were used for training (randomly selected). The classification performance was assessed by comparing the automatic and visual scoring of the fully manually annotated 4 d recordings of 43 mice. The global accuracy was computed using a confusion matrix^42^ of the completely predicted days (B1, B2, and R2). For all subsequent analyses, the visually annotated Day 3 (R1) recording and the algorithmically annotated days (B1, B2, and R2) were used for all mice, including those for which these days were visually annotated. The resulting sleep-wake state annotation together with EEG power spectra and EMG levels were saved as binary files (.smo) with their corresponding metadata files (.hdr) and deposited at Figshare^23^. For more information on .*smo* and .*hdr* files, see Usage Notes.

#### EEG/Behavioral Phenotyping

We quantified 341 phenotypes based on the sleep-wake states, LMA, and the spectral composition of the EEG, constituting 3 broad phenotypic categories. For the first phenotypic category (“State”), the 96 hours sleep-wake sequence of each animal was used to directly assess traits in 3 “state”-related phenotypic subcategories: (i) duration (e.g., time spent in wakefulness, NREM sleep, and REM sleep, both absolute and relative to each other, such as the ratio of time spent in REM versus NREM sleep); (ii) aspects of their distribution over the 24 h cycle (e.g., time course of hourly values, midpoint of the 12 h interval with highest time spent awake, and differences between the light and dark periods); and (iii) sleep-wake architecture (e.g., number and duration of sleep-wake bouts, sleep fragmentation, and sleep-wake state transition probabilities). Similarly, for the second phenotypic category (“LMA”) overall activity counts per day, as well as per unit of time spent awake, and the distribution of activity over the 24 h cycle was extracted from the LMA data. As final phenotypic category (“EEG”), EEG signals of the 4 different sleep-wake states (wakefulness, NREM sleep, REM sleep, and theta-dominated waking [TDW], see below) were quantified within the 4-s epochs matching the sleep-wake states using DFT (0.25 Hz resolution, range 0.75–90 Hz, window function Hamming). Signal power was calculated in discrete EEG frequency bands—i.e., delta (1.0–4.25 Hz, δ), slow delta (1.0–2.25 Hz; δ1), fast delta (2.5–4.25; δ2), theta (5.0–9.0 Hz during sleep and 6.0–10.0 Hz during TDW); θ), sigma (11–16 Hz; σ), beta (18–30 Hz; β), slow gamma (32–55 Hz; γ1), and fast gamma (55–80 Hz; γ2). Power in each frequency band was referenced to total EEG power over all frequencies (0.75–90 Hz) and all sleep-wake states in days B1 and B2 to account for interindividual variability in absolute power. The contribution of each sleep-wake state to this reference was weighted such that, e.g., animals spending more time in NREM sleep (during which total EEG power is higher) do not have a higher reference as a result^43^. Moreover, the frequency of dominant EEG rhythms was extracted as phenotypes, specifically that of the theta rhythm characteristic of REM sleep and TDW. The latter state, a substate of wakefulness, defined by the prevalence of theta activity in the EEG during waking^44,45^, was quantified according to the algorithm described in^46^. We assessed the time spent in this state, the fraction of total wakefulness it represents, and its distribution over 24 h. Finally, discrete, paroxysmal events were counted, such as sporadic spontaneous seizures and neocortical spindling, which are known features of D2 mice^47^, which we also found in some BXD lines.

All phenotypes were quantified in baseline and recovery separately, and the effect of SD on all variables was computed as recovery versus baseline differences or ratios. Pascal source code used for EEG/behavioral phenotyping was made available on gitlab (https://gitlab.unil.ch/mjan/Systems_Genetics_of_Sleep_Regulation). Processed phenotypes and descriptions were made available at https://bxd.vital-it.ch/#/dataset/1 and were submitted the Mouse Phenome Database^48^.

#### Read alignment

For gene expression quantification, we used a standard pipeline that was already applied in a previous study^6^. Bad quality reads tagged by Casava 1.82 were filtered from fastq files and reads were mapped to MGSCv37/mm9 using the STAR splice aligner (v 2.4.0 g) with the *2pass* pipeline^49^.

#### Genotyping

The RNA-seq dataset was also used to complement the publicly available GeneNetwork genetic map (www.genenetwork.org), thus increasing its resolution. RNA-seq variant calling was performed using the Genome Analysis ToolKit (GATK) from the Broad Institute, using the recommended workflow for RNA-seq data^50^. To improve coverage depth, 2 additional RNA-seq datasets from other projects using the same BXD lines were added^6^. In total, 6 BXD datasets from 4 different tissues (cortex, hypothalamus, brainstem, and liver) were used. A hard filtering procedure was applied as suggested by the GATK pipeline^50–52^. Furthermore, genotypes with more than 10% missing information, low quality (<5000), and redundant information were removed. GeneNetwork genotypes, which were discrepant with our RNA-seq experiment, were tagged as “unknown” (mean of 1% of the GeneNetwork genotypes/strain [0.05% ≤ n ≤ 8%]). Finally, GeneNetwork and our RNA-seq genotypes were merged into a unique set of around 11000 genotypes, which was used for all subsequent analyses. This set of genotypes was already used successfully in a previous study of BXD lines^6^ and is available through our “Swiss-BXD” web interface (https://bxd.vital-it.ch/#/dataset/1).

#### Protein damage prediction

Variants detected by our RNA-seq variant calling were annotated using Annovar^53^ with the RefSeq annotation dataset. Nonsynonymous variations were further investigated for protein disruption using Polyphen-2 version 2.2.2^54^, which was adapted for use in the mouse according to recommended configuration. Variant annotation file and polyphen2 scores were made available here^27^.

#### Gene expression quantification

Count data was generated using htseq-count from the HTseq (v0.5.4p3) package using parameters “stranded = reverse” and “mode = union”^55^. Gene boundaries were extracted from the mm9/refseq/reflat dataset of the UCSC table browser (extracted the 29^th^ Jan. 2014). Raw counts were made available^27^.

### Intermediate-level Layer Analyses

#### Gene expression normalization

EdgeR (v3.22) was then used to normalize read counts by library size. Genes with with low expression value were excluded from the analysis, and the raw read counts were normalized using the TMM normalization^56^ and converted to log counts per million (CPM). Although for both tissues, the RNA-seq samples passed all quality thresholds, and among-strain variability was small, more reads were mapped in cortex than in liver, and we observed a somewhat higher coefficient of variation in the raw gene read count in liver than in cortex. Genes expression as CPM or log2 CPM were made available^27^.

#### Differential expression

To assess the gene differential expression between the sleep-deprived and control conditions, we used the R package limma^57^ (v3.36) with the voom weighting function followed by the limma empirical Bayes method^58^. Differential expression tables were made available^27^.

#### QTL mapping

The R package qtl/r^33^ (version 1.41) was used for interval mapping of behavioral/EEG phenotypes (phQTLs) and metabolites (mQTLs). Pseudomarkers were imputed every cM, and genome-wide associations were calculated using the Expected-Maximization (EM) algorithm. p-values were corrected for FDR using permutation tests with 1000 random shuffles. The significance threshold was set to 0.05 FDR, a suggestive threshold to 0.63 FDR, and a highly suggestive threshold to 0.10 FDR according to^59,60^. QTL boundaries were determined using a 1.5 LOD support interval. To preserve sensitivity in QTL detection, we did not apply further p-value correction for the many phenotypes tested. Effect size of single QTLs was estimated using 2 methods. Method 1 does not consider eventual other QTLs present and computes effect size according to 1 − 10^(−(2/n)*LOD). Method 2 does consider multi-QTL effects and computes effect size by each contributing QTL by calculating first the full, additive model for all QTLs identified and, subsequently, estimating the effects of each contributing QTL by computing the variance lost when removing that QTL from the full model (“drop-one-term” analysis). For Method 2, the additive effect of multiple suggestive, highly suggestive, and significant QTLs was calculated using the fitqtl function of the qtl/r package^61^. With this method, the sum of single QTL effect estimation can be lower than the full model because of association between genotypes. In the Results section, Method 1 was used to estimate effect size, unless specified otherwise. It is important to note that the effect size estimated for a QTL represents the variance explained of the genetic portion of the variance (between-strain variability) quantified as heritability and not of the total variance observed for a given phenotype (i.e., within- plus between-strain variability).

For detection of eQTLs, cis-eQTLs were mapped using FastQTL^33^ within a 2 Mb window for which adjusted p-values were computed with 1000 permutations and beta distribution fitting. The R package qvalue^62^ (version 2.12) was then used for multiple-testing correction as proposed by^33^. Only the q-values are reported for each cis-eQTL in the text. Trans-eQTL detection was performed using a modified version of FastEpistasis^34^, on several million associations (approximately 15000 genes × 11000 markers), applying a global, hard p-value threshold of 1E−4.

List of ph-QTLs, cis-eQTL, trans-eQTL and m-QTLs were made available^27^.

### High-level layer Analyses

#### Hiveplot visualization

Hiveplots were constructed with the R package HiveR^63^ for each phenotype. Gene expression and metabolite levels represented in the hiveplots come from either the “Ctr” (control) or SD molecular datasets according to the phenotype represented in the hiveplot; i.e., the “Ctr” dataset is represented for phenotypes related to the baseline (“bsl”) condition, while the SD dataset is shown for phenotypes related to recovery (“rec” and “rec/bsl”). For a given hiveplot, only those genes and metabolites were included (depicted as nodes on the axes) for which the Pearson correlation coefficient between the phenotype concerned and the molecule passed a data-driven threshold set to the top 0.5% of all absolute correlations between all phenotypes on the one hand and all molecular (gene expression and metabolites) on the other. This threshold was calculated separately for “Bsl” phenotypes and for “Rec” and “Rec/Bsl” phenotypes and amounted to absolute correlation thresholds of 0.510 and 0.485, respectively. The latter was used for the recovery phenotypes. Associations between gene expressions and metabolites represented by the edges in the hiveplot were filtered using quantile thresholds (top 0.05% gene–gene associations, top 0.5% gene–metabolite associations). We corrected for cis-eQTL confounding effects by computing partial correlations between all possible pairs of genes. Hiveplots figures and Rmarkdowns reports were made available^27^.

#### Candidate-gene prioritization strategy

In order to prioritize genes in identified QTL regions, we chose to combine the results of the following analyses: (i) QTL mapping (phQTL or mQTL), (ii) correlation analysis, (iii) expression QTL (eQTL), (iv) protein damaging–variation prediction, and (v) DE. Each result was transformed into an “analysis score” using a min/max normalization, in which the contribution of extreme values was reduced by a winsorization of the results. These analysis scores were first associated with each gene (see below) and then integrated into a single “integrated score” computed separately for each tissue, yielding 1 integrated score in cortex and 1 in liver. The correlation analysis score, eQTL score, DE score, and protein damaging–variation score are already associated to genes, and these values were therefore attributed to the corresponding gene. To associate a gene with the ph-/m-QTL analysis score (which is associated to markers), we used the central position of the gene to infer the associated ph-/m-QTL analysis score at that position. In case of a cis-eQTL linked to a gene or a damaging variation within the gene, we used the position of the associated marker instead. To emphasize diversity and reduce analysis score information redundancy, we weighted each analysis score using the Henikoff algorithm. The individual scores were discretized before using the Henikoff algorithm, which was applied on all the genes within the ph-/mQTL region associated with each phenotype. The integrated score was calculated separately for cortex and liver. We performed a 10000-permutation procedure to compute an FDR for the integrated scores. For each permutation procedure, all 5 analysis scores were permutated, and a novel integrated score was computed again. The maximal integrated score for each permutation procedure was kept, and a significance threshold was set at quantile 95. Applying the Henikoff weighting improved the sensitivity of the gene prioritization. E.g., among the 91 behavioral/EEG phenotypes associated with 1 or more suggestive/significant QTLs after SD, 40 had at least 1 gene significantly prioritized with Henikoff weighting, against 32 without. Gene prioritization figures and Rmarkdown reports were made available^27^.

### Reproducibility of the pipeline

#### Technical reproducibility of the pipeline

To assess the reproducibility of our analytical pipeline, we asked a bioinformatician that was not involved in the data collection and analysis to reanalyze some of the results. A relatively short computational time as well as importance in the published results were taken as selection criteria of analyses to be replicated. The TMM normalisation of RNA-seq counts, differential gene expression, cis-eQTL detection, and the ph-/m-QTL mapping for 4 sleep phenotypes (slow delta power gain after SD, fast delta power after SD, theta peak frequency shift after SD and NREM sleep gain in the dark after SD) and 2 metabolites (Phosphatidylcholine ae C38:2 and alpha amino-adipic acid) used as main examples in our previous publication were all re-analyzed. Finally, gene prioritization and hiveplot visualization of these 4 examples were replicated. Originally, ties in the nodes ranking function on the hiveplots axis was solved using the “random” method, but this function was modified in the hiveplot code and set as “first” to remain deterministic (see Technical Validation for results).

#### Reanalysis with mm10

To quantify the effect of new standards and robustness of our end-results and interpretation we changed some analyses within our low-level layer. The mm10 genome assembly was set as our new reference and the gene expression was reanalysed from the raw fastq files with the BioJupies reproducible pipeline^64,65^ that use kallisto pseudo-alignement^66^. The gene positions were retrieved from the headers of the ENSEMBL fasta file used by BioJupies (Mus_musculus.GRCm38.cdna.all.fa.gz). Genotypes were downloaded from GeneNetwork database and our annovar/polyphen2 variations positions based on mm9 were adapted to mm10 using CrossMap version 0.2.4^67^. The analyses performed to assess the technical reproducibility of our pipeline (see above) were finally replicated using these new files. (see Technical Validation for results).

## Data Records

EEG/EMG power spectra and locomotor activity files were submitted to Figshare^23^. Raw data of RNA-sequencing were submitted to Gene Expression Omnibus^24^. Processed phenotypes files as gene expression, metabolites level and mean EEG/behavioral phenotypes per lines, as well as phenotypes descriptions, were submitted to our data-mining web-site (https://bxd.vital-it.ch/#/dataset/1) on the ‘Downloads’ panel. Scripts and code were submitted to gitlab (https://gitlab.unil.ch/mjan/Systems_Genetics_of_Sleep_Regulation). Intermediate files required to run these scripts were submitted to *Figshare*^27^. The data hosted on our server and the data we used from external repositories like *GeneNetwork* original genotypes^40^ and RefSeq transcripts^68^ were also copied on Figshare^27^ for reproducibility purpose. Please cite R.Williams or the NCBI if you use these two files.

## Technical Validation

### Compare genotype RNA-seq vs GeneNetwork

**Fig. 4:**
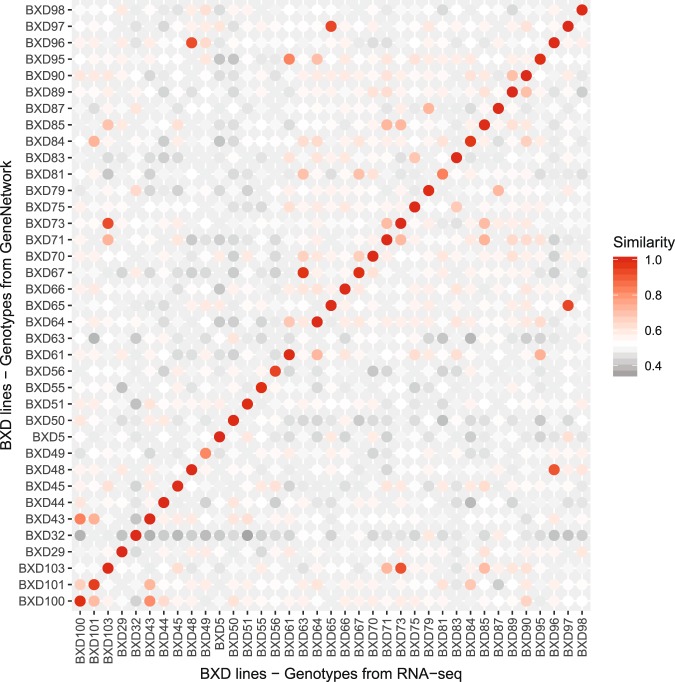
Similarity matrix [in %] between RNA-seq variant calling and GeneNetwork genotypes. A similarity of 1 indicates that all common genotypes are similar. We here compare only genotypes that were labeled as ‘B’ or ‘D’ and excluded unknown ‘U’ or heterozygous ‘H’ genotypes. BXD63 genotypic similarity in our dataset was low and could indicate mislabeling.

To verify the genetic background of each mice we phenotyped, we analyzed the correspondence between GeneNetwork genotypes and RNA-seq variants detected by GATK. Of the 3811 GeneNetwork (2005) genotypes, 1289 could be recalled in our RNA-seq variant calling pipeline. Figure 4 shows the similarity proportion between RNA-seq variants and GeneNetwork genotypes, for each pair of BXD lines. Our BXD63 was more similar with the GeneNetwork BXD67 than with the BXD63, probably due to mislabeling. We therefore chose to exclude this line. The matrix also shows the genetic similarity between BXD73 and BXD103 (now renamed as BXD73b), between BXD48 and BXD96 (now BXD48a) and between BXD65 and BXD97 (now BXD65a), which confirmed the renaming of these BXD lines on GeneNetwork.

### Reproducibility of the pipeline

#### Technical reproducibility of the pipeline

**Fig. 5:**
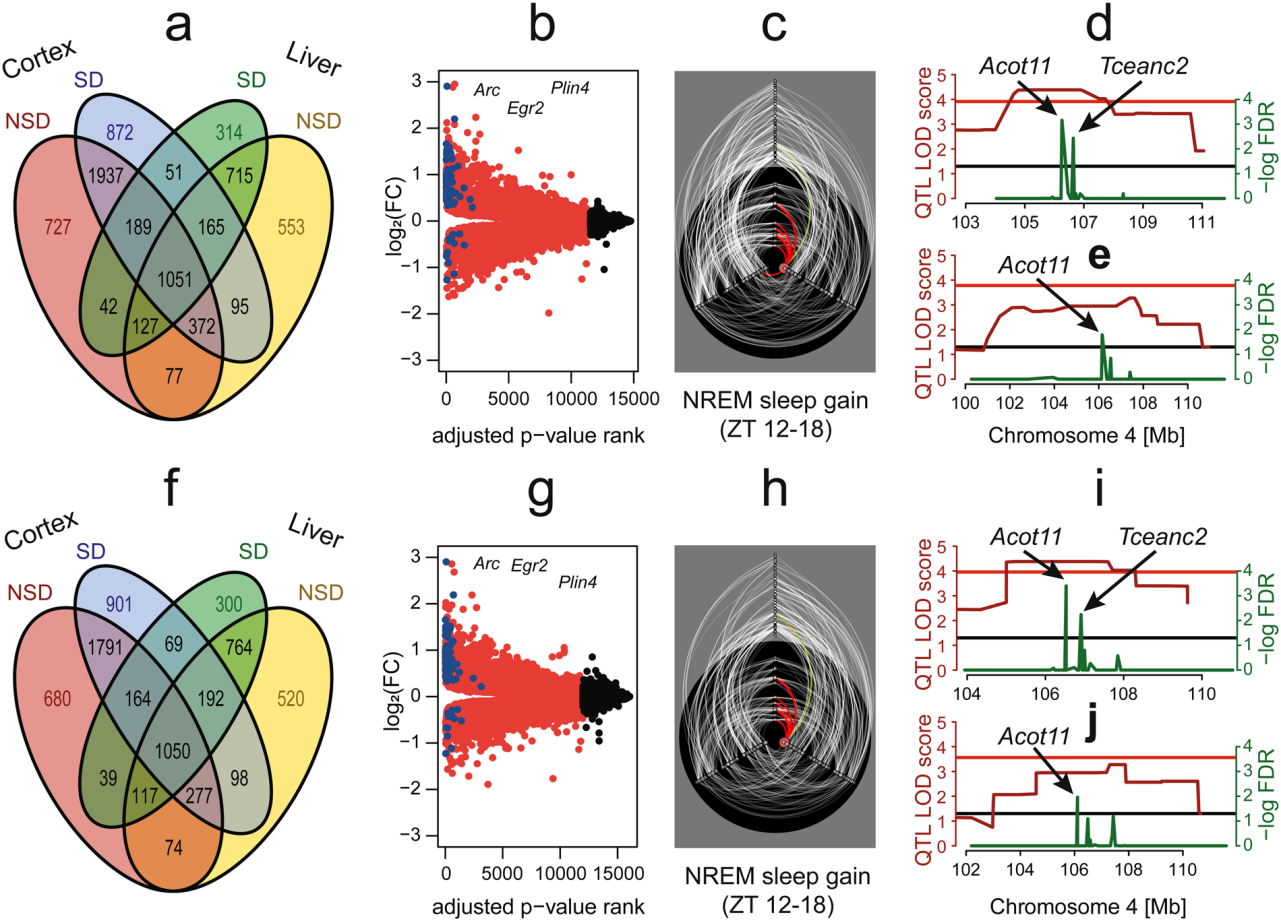
Robustness of the analysis pipeline. (**a to e**) Technical reanalysis with mm9 reference genome. (**f to j**) Reanalysis with mm10 reference genome. (**a and f**) Venn diagram of significant cis-eQTL. (**b and g**) Volcano plot of differential gene expression in cortex. (**c and h**): Hiveplot for NREM sleep gain during recovery with highlight on *Acot11*. (**d,e,i,j**) Gene prioritization for NREM sleep gain during recovery (**d and i**) or phosphatidylcholine acyl-alkyl C38:2 levels (**e and j**). recovery = first 6 hours of dark period after sleep deprivation (ZT 12–18), SD = sleep deprivation, NSD = not sleep deprivation (control), FC = fold-change, NREM = non-rapid eye movement, LOD = logarithm of odds, FDR = false discovery rate.

To assess the technical reproducibility of the pipeline, a bioinformatics student (NG) new to the project, reproduced selected steps of the bioinformatic pipeline. The results (Fig. 5, upper part) were consistent with previous analyses (PLOS Biology publication figures: 2c, 4c left, 7d, and 7c bottom). The robustness of the pipeline was verified because the same conclusions could be drawn. For examples, the same 3 genes showed the largest differential expression after SD in the cortex (*Arc*, *Plin4*, and *Egr2* in Fig. 5b). Moreover, the *Acot11* gene was prioritized by gene prioritization (Fig. 5 d and e). Nevertheless, the numbers of significant genes of cis-eQTL showed variations compared to previous analysis^2^ due to use of a hard significance threshold for visualization. For example, the number of genes with significant QTL unique to Cortex SD changed from 870 (PLOS Biology publication Fig. 2c) to 872 (Fig. 5a). Genes were considered as significant if their FDR-adjusted p-value was below or equal to 0.05, which was obtained by estimating the β-distribution fitting of random permutations tests. Changing the fastqtl version (version 1.165 to version 2.184) seems to change the pseudo-random number generation, even when using the concept of fixed seed. Consequently, the number of genes considered as significant varies because their FDR-adjusted p-value passed just above or below the threshold (FDR in the range of 0.04864 to 0.05054). This confirms that looking at the order of magnitude is important, though the use of significance threshold is convenient.

Moreover, the reanalysis process helped to improve the code documentation by explicitly writing project-related knowledge, such as common abbreviations. Having another perspective on the code also allowed to improve its structure. Indeed, a retrospective overview helped improve the organisation of files, which was more difficult to do within the implementation phase of the project because the code was incrementally created and adapted. The process allowed to catch and correct minor mistakes or make improvements to readability and consistency. For example, it was highlighted that the ranking function used in hiveplot to order nodes in the axes was using the “random” argument for differentiating ties. As a key concept of the hiveplots was to be fully reproducible in the sense of “perpetual uniformity”^63^, we changed the ties.method parameter to “first” so that the same input always gives the same result, without having to fix a seed for the pseudo-random generation. Another example was the ranking of the x-axis in the gene DE volcano plot and the colouring that were based on log-odds values (B statistic according to in limma R package) instead of FDR-adjusted p-values. However, this reproducibility ‘experiment’ was performed internal to the group, which facilitated communication such as which steps to focus on and whether to run them locally or on a high-performance computing (HPC) structure. An assessment of the computational requirements for each step, such as computing time, memory, software, and libraries used may be interesting to provide to facilitate external reproducibility.

#### Reanalysis with mm10

To assess the influence of the reference genome used in the analyses, we reproduced selected parts of bioinformatic pipeline using the updated mm10 version (instead of mm9). The results (Fig. 5, lower part, Tables 1 and 2) were consistent with previous analyses but presented also some substantial variations. The cis-eQTL detection revealed differences in the number of significant associations found, as showed in Table 1. These differences could be mainly explained by small q-value variation around the significant threshold. Nevertheless, around 5% of cis-eQTLs did not reproduce even at a more permissive significant threshold (0.1 FDR), which affected some of our end results. For example, *Wrn* was no longer prioritized for the gain of slow EEG delta power (δ1) after SD compared to previous results on mm9. Although the cis-eQTL for *Wrn* was present in both assemblies for the ‘Cortex Control’ samples, it disappeared for ‘Cortex SD’ samples using mm10. A number of factors could have contributed to this discrepancy among which i) the variations between mm9 and mm10 could change the mappability of some transcripts, although this did not seem to be the case for the *Wrn* sequence, ii) pseudo-alignment (Kallisto) was used instead of alignment (STAR), which may have influenced the quantification, iii) bad quality reads were filtered with our STAR pipeline according to Casava 1.82 but not with Kallisto, and iv) variant calling on RNA-seq data to add markers was not done for mm10, so only markers from GeneNetwork (2017) were used. Specifically to the latter factor, the marker closest to the *Wrn* gene in mm9 merged (GeneNetwork 2005 + RNA-seq) genotypes (rs51740715) is not present in mm10. The change in the number of genetic markers could have therefore influenced the cis-eQTL detection, which is an important factor in the gene prioritization that resulted in the identification of *Wrn* as candidate underlying the EEG delta power (δ1) trait under mm9.

**Table 1:** Comparison of cis-eQTL summary statistics using mm9 vs mm10.

Assembly	Liver NSD		Liver SD		Cortex NSD		Cortex SD	
	mm9	mm10	mm9	mm10	mm9	mm10	mm9	mm10
Total genes	14103	12647	14103	12647	14889	15734	14889	15734
Unique genes	2405	949	2405	949	1043	1888	1043	1888
Genes with significant cis-eQTL	3155	3092	2654	2695	4522	4192	4732	4542
Proportion of genes with significant cis-eQTL	0.22	0.24	0.19	0.21	0.30	0.27	0.32	0.29
Genes with significant cis-eQTL overlapping	2255		1911		3204		3483	
Genes with not significant cis-eQTL overlapping	8375		8857		9062		8801	
Genes with significant cis-eQTL not overlapping	900	837	743	784	1318	988	1249	1059
Genes with significant cis-eQTL almost overlapping	2995	2898	2535	2505	4201	4019	4441	4350

‘Unique’ is defined as specific to an assembly (mm9 or mm10). Significance is defined as a q-value below or equal to 0.05. ‘Overlapping’ is defined as common between mm9 and mm10 reanalyses. ‘Almost overlapping’ is defined as common between mm9 and mm10 at a threshold of 0.1 but not as the 0.05 threshold.

**Table 2:** Comparison of gene DE in mm9 and mm10 reanalyses. Suggestive is defined as a q-value below or equal to 0.1.

	Liver		Cortex	
	mm9	mm10	mm9	mm10
Total genes	12539	13264	14754	16057
DE genes	7573	8754	11534	11980
Proportion of DE genes	0.6040	0.6600	0.7818	0.7461
Suggestive DE genes	8253	9392	12069	12580
Proportion of suggestive DE genes	0.6582	0.7081	0.8180	0.7835

## Usage Notes

### SMO files

Binary .*smo* files were structured as follows: Each file contains a 4-day recording or precisely 86400 consecutive 4 s epochs. Each 4 s epoch contains the following information: one byte character and 404 single precision floating-points, which represent, respectively: sleep-wake state of the 4 s epoch as a character (wake = ‘w’, NREM sleep = ‘n’, REM sleep = ‘r’, wake w/ EEG artifact = ‘1’, NREM sleep w/ EEG artifact = ‘2’, REM sleep w/ EEG artifact = ‘3’, wake w/ spindle-like EEG activity = ‘4’, NREM sleep w/ spindle-like EEG activity = ‘5’, REM sleep w/ spindle-like EEG activity = ‘6’, Paroxysmal EEG activity in wake = ‘7’, Paroxysmal EEG activity in NREM sleep = ‘8’, Paroxysmal EEG activity in REM sleep = ‘9’), EEG power density from the full DFT spectrum of the 4 s epoch from 0.00 Hz to 100.00 Hz (401 values at 0.25-Hz resolution), the EEG variance, the EMG variance, and temperature. Temperature was not measured and was set to 0.0.

### HDR files

Text .*hdr* files are generated alongside their corresponding .*smo* file and contain among other information, the mouse ID (*Patient*) and recording date.

### Rmarkdown scripts

Some of the Rmarkdown scripts were created for a remote cluster environment on a CentOS distribution which required the use of a second script that generated the document with the *rmarkdown::render()* function and pass the expected function arguments. Therefore some functions that use the parallel package in R are only executable on a linux environment (i.e. mclapply()). These functions can be modified with the *doSNOW* R library to be applicable on a windows environment. The author can set many option in the YAML (Yet Another Markup Language) header to: create dynamic and readable table that contains multiple rows, hide/show source code or integrated CSS style and table of contents. The reports can be visualized using any web-browser.

### ISA-Tab metadata file

Download metadata file

## Data Availability

The scripts used for analytics were made available on gitlab (https://gitlab.unil.ch/mjan/Systems_Genetics_of_Sleep_Regulation). The master branch contains the scripts used for our publication and mm9 analysis. A second branch was created for analysis performed on a mm10 mouse references (see Technical Validation). The intermediate files required to run these scripts were made available at Figshare^27^. Finally, a documentation file was generated documenting the hierarchical relationship between the scripts and datasets in a form of a dynamic html document (see Workflow documentation).

## Online-only Table

**Online-only Table 1:** Names of BXD lines used in our files with the corresponding JAX name and ID. F1 lines (DB and BD) were generated in house. BXD line names in our files can also be found without ‘0’ i.e. BXD50 instead of BXD050. Further note that the names we used followed an older nomenclature and some names therefore differ from the current JAX names listed.

Name in files	JAX Name	JAX ID
BXD005	BXD5/TyJ	37
BXD029TL/BXD029t	BXD29- Tlr4^lps-2J^/J	29
BXD029	BXD29/Ty	10981
BXD032	BXD32/TyJ	78
BXD043	BXD43/RwwJ	7093
BXD044	BXD44/RwwJ	7094
BXD045	BXD45/RwwJ	7096
BXD048	BXD48/RwwJ	7097
BXD049	BXD49/RwwJ	7098
BXD050	BXD50/RwwJ	7099
BXD051	BXD51/RwwJ	7100
BXD055	BXD55/RwwJ	7103
BXD056	BXD56/RwwJ	7104
BXD061	BXD61/RwwJ	7106
BXD063	BXD63/RwwJ	7108
BXD064	BXD64/RwwJ	7109
BXD065	BXD65/RwwJ	7110
BXD066	BXD66/RwwJ	7111
BXD067	BXD67/RwwJ	7112
BXD070	BXD70/RwwJ	7115
BXD071	BXD71/RwwJ	7116
BXD073	BXD73/RwwJ	7117
BXD075	BXD75/RwwJ	7119
BXD079	BXD79/RwwJ	7123
BXD081	BXD81/RwwJ	7125
BXD083	BXD83/RwwJ	7126
BXD084	BXD84/RwwJ	7127
BXD085	BXD85/RwwJ	7128
BXD087	BXD87/RwwJ	7130
BXD089	BXD89/RwwJ	7132
BXD090	BXD90/RwwJ	7133
BXD095	BXD95/RwwJ	7138
BXD096	BXD48a/RwwJ	7139
BXD097	BXD65a/RwwJ	7140
BXD098	BXD98/RwwJ	7141
BXD100	BXD100/RwwJ	7143
BXD101	BXD101/RwwJ	7144
BXD103	BXD73b/RwwJ	7146
C57BL6/B61/B62	C57BL/6 J	664
DBA2/DB1/DB2	DBA/2 J	671
DB/DXB F1/DBA/2 J x C57BL6/J F1	—	—
BD/BXD F1/C57BL6/J x DBA/2 J F1	—	—
